# The more you do it, the easier it gets: using behaviour change theory to support health care professionals offering reproductive genetic carrier screening

**DOI:** 10.1038/s41431-022-01224-5

**Published:** 2022-11-24

**Authors:** Stephanie Best, Janet C. Long, Zoe Fehlberg, Tahlia Theodorou, Sarah Hatem, Alison Archibald, Jeffrey Braithwaite

**Affiliations:** 1 Australian Institute of Health Innovation, Macquarie University, Sydney, NSW Australia; 2 Australian Genomics, Murdoch Children’s Research Institute, Melbourne, VIC Australia; 3 Dept of Health Services Research, Peter MacCallum Cancer Centre, Parkville, Australia; 4 Dept of Health Services Research, Victorian Comprehensive Cancer Centre Alliance, Parkville, Australia; 5 Sir Peter MacCallum Dept of Oncology, University of Melbourne, Parkville, Australia; 6 Bruce Lefroy Centre, Murdoch Children’s Research Institute, Royal Children’s Hospital, Melbourne, VIC Australia; 7 Department of Paediatrics, Faculty of Medicine, Dentistry and Health Sciences, University of Melbourne, Melbourne, VIC Australia

**Keywords:** Genetic testing, Population screening

## Abstract

Recent advances in genomic sequencing have improved the accessibility of reproductive genetic carrier screening (RGCS). As awareness and interest grows, non-genetic health care professionals are increasingly offering RGCS to consumers. We conducted a qualitative interview study informed by behaviour change theory to identify influences on health care professionals considered as ‘early adopters’ offering RGCS through Mackenzie’s Mission, an Australian national research study investigating the implementation of free RGCS to couple’s preconception or in early pregnancy. Interviews were deductively analysed using the Theoretical Domains Framework to examine barriers and enabling factors. In total, we interviewed 31 health care professionals, who were primarily general practitioners (*n* = 23) offering RGCS through Mackenzie’s Mission. Upon analysis, 15 barriers and 44 enablers to implementation were identified and categorised across three health care professional target behaviours 1. Engaging with RGCS, 2. Identifying eligible patients, and 3. Offering RGCS. Whilst all Theoretical Domains Framework domains were present, barriers were predominantly categorised as ‘Environmental Context and Resources’ e.g., lack of time, followed by ‘Knowledge’ e.g., lack of understanding about genetics and ‘Beliefs about Capabilities’ e.g., concern about giving high risk results to patients. Although health care professionals expressed a preference for offering RGCS through a comprehensive and supported model of care, such as Mackenzie’s Mission, barriers remain. By understanding what drives current health care professionals’ behaviour towards offering RGCS, behaviour change theory provides an avenue to direct future efforts based on evidence and improve service delivery.

## Introduction

Reproductive genetic carrier screening (RCGS) allows prospective parents to gain knowledge of their chance of having children with a serious autosomal-recessive or X-linked genetic condition. When a couple are both found to be carriers of a condition, they have an ‘increased chance’, of having an affected child [1]. As such, screening is offered preconception or in early pregnancy to facilitate greater reproductive choices [2]. Recent advances in technology have increased affordability of RGCS moving from single-gene testing e.g., Tay-Sachs disease [3] to allowing screening for multiple conditions (i.e., ‘expanded’ screening) driving international recognition of the benefits to population wide screening [4, 5]. In Australia, RGCS is predominantly available commercially to individuals or couples, however the cost and lack of public and medical practitioner awareness of screening exacerbates inequities of access and outcomes across populations [6, 7]. In response, and to reflect changing practice guidelines, some national health systems (e.g., Australia, Belgium) have started investigating population level RGCS [3].

Expanding the availability of RGCS requires non-genetic health care professionals e.g., general practitioners (GPs), obstetricians, fertility specialists and midwives to play an important role in offering RGCS. Decades of single-gene screening have provided a rich foundation of knowledge about health care professionals’ (HCPs’) perceptions towards offering population-based screening for individual conditions [8–10]. However, research examining expanded carrier screening has been limited primarily to genetic HCP or secondary HCP perspectives e.g., gynaecologists and obstetricians [11, 12] or focused on hypothetical offering in the primary healthcare setting [13, 14]. A recent review identified a predominance of practitioner level barriers (i.e., lack of practitioner confidence, interest) and organisational level enablers (i.e., professional bodies providing consistent advice) [15].

**Fig. 1:**
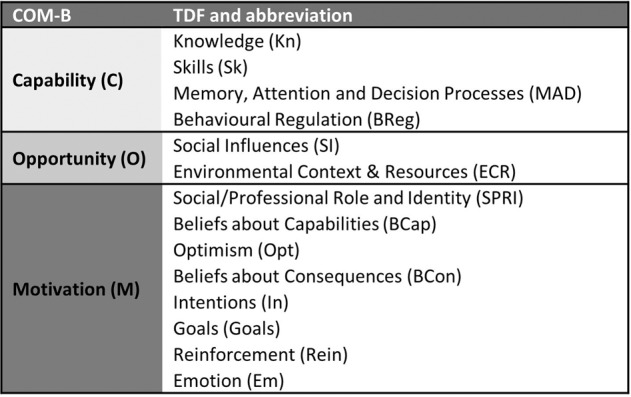
Alignment of Capability, Opportunity and Motivation, Behaviour (COM-B) and Theoretical Domains Framework (TDF) with abbreviations.

An in-depth understanding of this area is hampered by a lack of targeted implementation research examining the implementation of RGCS at a population scale. The introduction of new practices, such as offering RGCS, requires a change in practice of the HCPs. Behaviour change theory provides a way to analyse what is driving current behaviours (i.e., current practice) of HCPs and identify interventions that support the new desired behaviour [16]. Theory informed frameworks such as the ‘Capability, Opportunity, Motivation and Behaviour’ (COM-B) model and associated Theoretical Domains Framework (TDF) are effective for understanding contextual influences on desired behaviour [17]. The COM-B framework posits that behaviour is a result of our capability (C) (can we do the activity), opportunity (O) (is it possible to do it) and our motivation (do we want to do it) (M). The TDF consists of 14 domains and provides a more granular understanding of the influences on behaviour. For example, several TDF domains influence motivation (e.g., Emotion, and Belief about Consequences) and therefore align with the COM-B domain motivation (Fig. 1).

In this study, we use qualitative methods informed by behaviour change frameworks, to investigate the experience of early adopters of RGCS in the context of a national research programme. Our objective is to gain an insight into HCPs’ perceived barriers and enablers to offering RGCS.

## Materials and methods

### Context

This study is part of a larger implementation science programme investigating strategies to support RGCS implementation in Mackenzie’s Mission. Mackenzie’s Mission is an Australian Government funded research study investigating how to optimally implement an easily accessible RGCS programme in Australia by offering couples free RGCS, testing over a thousand genes [18]. Details of the study are published elsewhere [18] but briefly couples were directed to the Mackenzie’s Mission website via an initial conversation with their GP, obstetrician, midwife, fertility specialist or genetics HCP. The portal contained information, a decision support tool and how to register. If the couple proceeded, they collected and posted in a cheek swab. HCPs were not included in this process but were informed of results.

### Research design

We employed a qualitative approach using semi-structured interviews with a subset of participating HCPs to gain a rich understanding of the meanings and interpretations behind individuals’ behaviour towards offering RGCS through Mackenzie’s Mission [19]. We adopted descriptive phenomenology which can be used to study an area of interest from the perspective of those involved [20], using behaviour change theory as our methodological framework.

### Participants and recruitment

Throughout the study duration, HCPs from various settings in all Australian states and territories (including general practices, private/public obstetrics and midwifery practices, fertility clinics, and community health services) were able to self-refer into the Mackenzie’s Mission study or were invited by study genetic counsellors. As the focus of the study was on preconception RGCS, the intention was for most couples to be recruited via GPs. HCPs who agreed to be part of Mackenzie’s Mission were provided with an education session about RGCS and the study processes before being able to offer Mackenzie’s Mission RGCS to their patients.

HCPs who expressed interest via a pre-education questionnaire *and* had been offering Mackenzie’s Mission for >8weeks were contacted via email, by the implementation research team, and invited to take part in either a telephone or videoconference interview. One follow-up invitation was sent for those who did not respond to the initial contact. Using purposive sampling [21], we selected over 150 eligible HCPs (*n* = 168) to invite to take part in an interview. HCPs were from a range of professions and levels of experience of RGCS, in particular GPs as the predominant referrers, and different state/territories. Those HCPs who declined to participate in the Mackenzie’s Mission study were also offered an opportunity to interview.

### Data collection tools and procedures

An interview guide (Supplementary File 1) was developed using the behavioural framework COM-B, as a way to categorise sources of behaviour [22]. For example, ‘*what experience do you have with RGCS?*’ (C); ‘*Starting off, was there anything that would have made offering RGCS easier?*’ (O); and ‘*What made you decide to offer RGCS?*’ (M). Participants were asked about their experiences of offering RGCS and their views on future RGCS service planning. Whilst the structure of the guide remained the same, constant iterative comparison [23] of the interview transcripts led to minor revisions in the interview schedule. Interviews were designed to take around 30 min, and scheduled at a time convenient for the HCP. Interviews were disrupted by the SARS-CoV-2 pandemic and began in August 2020, finishing in August 2021 when data saturation and maximum variation in sample was reached. Interviews were conducted by three qualitative researchers (SB, JL and ZF) who had no prior relationship with participants. All interviews were audio-recorded, de-identified and transcribed verbatim. Audio-recorded verbal consent was sought and recorded before the interview.

### Data analysis

Interview data was managed in NVivo 12 [24]. Analysis was guided by the TDF. A coding guide that incorporated the TDF was adapted to the specific context (Supplementary File 2), revised from a previously published guide [25]. An important first step to using the TDF is to define the target behaviour i.e., the core activity that is essential for a change in practice to occur. For this study ‘*offering RGCS*’ was identified. However, during analysis, two additional target behaviours became evident. 1. Engaging with RGCS—how HCPs initially start thinking about offering RGCS; 2. Identifying eligible patients to offer RGCS to—including pre-conception; and finally the original target behaviour, 3. Offering RGCS to patients—incorporates the discussion with potential patients their perceived receptivity and following required process. Transcripts were examined and deductively coded using the coding guide (Supplementary File 2) to identify factors that facilitate or hinder HCPs when offering RGCS. Initially, five transcripts were coded independently by two researchers (SB and ZF) and compared for discrepancies. One researcher (ZF) completed the coding with ongoing regular meetings (SB and JL) to discuss and resolve challenging coding and findings. Reflecting the complexity of offering RGCS, overarching barriers were identified first before detailing the underlying barriers and determiningtheir associated TDF coding (SB, ZF and JL).

## Results

Participant characteristics are presented first, followed by the analysis of the three target behaviour barriers and enablers and associated TDF codes.

### Characteristics of participants

Overall, ~1000 HCPs were enroled in the Mackenzie’s Mission study. Of the 168 eligible HCPs invited to an interview, thirty-one agreed. The few participants who actively declined and gave reasons noted they were too busy especially because of the SARS-CoV-2 pandemic or they had not offered RGCS due to seeing different patient cohorts. No HCP who declined to participate in Mackenzie’s Mission indicated they were available for a follow-up interview. On average interviews ran for 24 min most were undertaken via videoconference and one participant opted for a telephone interview. Table 1 summarises the characteristics of interview participants who were predominantly GPs (74%) working in metropolitan areas of Australia (84%) with a fair proportional distribution amongst states per population size. Most participants had prior experience of offering RGCS (68%) and eight (26%) had experienced a patient receive an increased chance result as part of the Mackenzie’s Mission study.

**Table 1:** Sample characteristics.

HCP characteristics (*n* = 31)	*n* (%)
Gender	
Female	26 (83.87)
State	
New South Wales	11 (35.48)
Queensland	8 (25.81)
Western Australia	5 (16.31)
Victoria	4 (12.90)
South Australia	3 (9.68)
Region	
Metropolitan	26 (83.87)
Inner regional	3 (9.68)
Outer regional	2 (6.45)
Remote	0
Role	
GP	23 (74.19)
Midwife	3 (9.68)
Clinical Geneticist	4 (12.90)
Sexual Health Nurse	1 (3.23)
Prior experience with RGCS	
Yes	21 (67.74)
Had patient with an increased chance result	
Yes	8 (25.80)

### Barriers and enabling factors by target behaviour

**Fig. 2:**
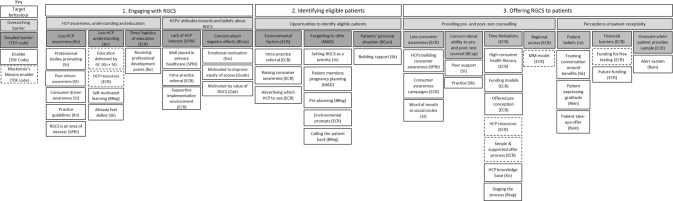
Overview of target behaviours, barriers and enablers to offering reproductive genetic carrier screening. Abbreviations: (Kn) Knowledge, (ECR) Environmental Context and Resources, (SPRI) Social Professional Role and Identity, (BCon) Beliefs about Consequences, (MAD) Memory, Attention and Decision Making, (SI) Social Influences, (Sk) Skills (Breg) Behaviour Regulation, (Rein) Reinforcement, (Em) Emotion, (Opt) Optimism, (In) Intentions.

From the interviews, 15 barriers and 44 enablers (6 of which related to specific programme components of Mackenzie’s Mission) were identified across the three target behaviours 1. Engaging with RGCS, 2. Identifying eligible patients to offer RGCS to, and 3. Offering RGCS to patients. Whilst all TDF domains were present, barriers were predominantly categorised as ‘Environmental Context and Resources’ followed by ‘Knowledge’ and ‘Beliefs about Capabilities’ (Fig. 2). Here, we outline the barriers and report recurrent enablers as reported by participants, further details are reported in Tables 2–4.

**Table 2:** Detailed summary Target behaviour 1: Engaging with RGCS.

HCP awareness, understanding and education			
Barrier	Exemplar quotes	Enabler	Exemplar quotes
**i Low HCP awareness of RGCS** TDF: Knowledge	I would say not a lot of GPs are aware that this was available, like the two GPs in my clinic were not aware that this was available even. GP24	**Professional body(ies) promoting RGCS** TDF: Social Influences	I wasn’t all that familiar with it, and it wasn’t until I did go to an Obs and Gynae workshop and they were talking about the different companies that provide it and that we should be offering it to everybody, and I was thinking that I was bit behind the eight-ball on this. GP29 It helps when the College of Obstetricians has made statements that this should be discussed with all couples or in early pregnancies. I guess I am just hoping that word of mouth practice just becomes part of the vernacular. CG17
		**Peer(s) promoting and building RGCS awareness** TDF: Social influences	One of the geneticists told me or it was at a [local] health day…that we were obliged as health care practitioners to offer it [RGCS] or to increase awareness for families. MW08
		**Consumer driven awareness** TDF: Social influences	One of my patients had brought it [RGCS] up with me, which was one of the reasons why I thought I would be interested to do it [MM]. GP30
		**HCP awareness of relevant practice guidelines** TDF: Knowledge	The first time I read about it was the RACGP guideline, they said all GPs should offer it, so it was like, oh gosh! So I read it about four times and then started offering it, but I think it took up to maybe two years after that until all my colleagues sort of clued in. GP13
		**RGCS is an area of interest** TDF: Social Professional Role and Identity	Because it is one of my interests of doing women’s health and family planning, I sort of knew about it then. GP24
**ii Low underlying HCP genetics and RGCS knowledge** TDF: Knowledge	…thinking I don’t know too much about it [RGCS] and how am I going to be able to offer this service when I know nothing about it? GP19	**RGCS education** TDF: Social Influences and Knowledge	I found that [education session] really, really helpful. It was very educational for me. I was pretty open to [the GC] initially saying, ‘look, my knowledge of this is pretty poor now because I haven’t done genetics properly since medical school’ but probably had a little bit more awareness than I thought I did. GP27 I personally learned a lot. I had so many questions to ask… and the education did provide me that education, and I was able to clearly reiterate that information to the patients, I think that was my biggest concern. MW10
		**Resources for HCPs (GC support, peers, website and informative notes)** TDF: Environmental Context and Resources	I very much felt I had the support of the research lady here [genetic counsellor]. And I knew she was going to be able to help me out if I knew nothing about it. GP19 One of the staff members has done a little two-page thing for other staff members just to make it nice and clear. GP22 I think there was a lot of information there [MM website] so I didn’t feel there was a lot of trouble understanding it all or the recruitment process. GP24
		**Self-directed learning** TDF: Behaviour Regulation	I did some reading up on this immediately and then ingrained in my mind what were the exact differences [NIPS and RGCS]. GP06 I was doing some reading up and became aware of the study, because I felt that was a gap in my knowledge. Obviously, I was offering all the antenatal screening, apart from the genetic carrier screening which is now something that I do mention to couples. GP12
		**Already feeling skilled at offering RGCS** TDF: Skills	I’m already quite used to talking about offering that screening. And also, patients don’t expect me to have all the answers GP05
**iii Time constraints attending RGCS education session** TDF: Environmental Context and Resources	I think the main barrier for them (colleagues) would be time constraints, same with me, so initially the training was an hour zoom meeting, which was doableGP11	**Receiving professional development points for completing education** TDF: Behaviour Regulation	If it was a CPD activity and you could get points back for education I think that would be more attractive. GP11
		**Flexible modes of education** TDF: Environmental Context and Resources	We could watch the [education] videos when it suited us, so whether that be during the day or at night, that was up to us. SHNurse20

HCPs’ attitudes towards and beliefs about RGCS			
**i RGCS not an interest or priority or is not in HCP’s scope of practice** TDF: Social Professional Role and Identity	Professionally, not a lot of midwives would understand, or maybe see the need for pregenetic testing because they just see the normal births that come through and don’t realise the complications. MW04 Not every GP should have to do it because if someone does it, they should probably do it well, and they should probably know the landscape of different offerings, as opposed to, ‘this is the only test I know, and it will cost you $1000’ GP13 In my practice of around eight doctors, I’m the main provider of antenatal care so I find it can be tricky a little bit when your colleagues don’t have the same interest too, so the levels of confidence and experience in providing reproductive carrier screening counselling varies quite a lot throughout GPs and they’re the main group of people that I have regular contact with. GP11	**Well-placed in primary healthcare** TDF: Social Professional Role and Identity	It fits in so nicely with our current focus on preconception counselling, which I think is the domain of the GP and targeting the woman even before she starts embarking on her reproductive course. I think the GPs are the ones who should be actually focusing on this and providing this, and it comes naturally to us…I think to make it really successful it should be in primary care, and the RACGP guidelines they are actually encouraging us to offer it, so they’ve embraced it, so as primary care providers we should be embracing it. GP06
		**Other professions who could offer or be aware of RGCS** TDF: Social Professional Role and Identity	We [clinical geneticists] can be later on, we can be a second tier and we can tell them this is available, but it should actually come from a grass root level because that is where you would be capturing most of the population, we see a very niche population. So, for it to be most effective, it should be started at the level of the GPs. We can always be involved. CG16 You could certainly target fertility clinics, practise nurses are a good idea actually because I think that GPs that have practise nurses, that could definitely be in their scope. If you are looking to bring it into nursing and midwifery the gyn nurses, but midwifery itself, no. MW08 School nurses should be involved like the Jewish screening programme. CG09 I’m a no door is the wrong door kinda gal. I don’t care if you are a midwife, I don’t care if you are an obstetrician, a GP, oh for goodness sake let the pharmacist do it. GP25
		**Intra-practice referral** TDF: Environmental Context and Resources	The males do offer it as well, just some of them, if they’re a bit uncomfortable, or unsure, or it’s not their area of interest, at least they know about it, and they’ll often refer across to us. GP13 We have very collaborative practice. So as soon as I had the forms in, I could email everyone who does shared care and let them know that I was able to offer it. And we’ve been able to work really collaboratively within our practice for that to happen. GP26
		**Supportive implementation environment** TDF: Environmental Context and Resources	We were just ready for it, so genetic services were happy to recruit. And I guess also fortunately our laboratory was keen to be involved. CG17
**ii HCP concern about possible negative consequences (anxiety, financial implications)** TDF: Beliefs about Consequences	The guy I used to work for, an obstetrician would say ‘oh it’s such a wretched thing to talk about, screening for Down Syndrome, you’ve got a young keen person all excited about pregnancy and you have to talk about all these things that could go wrong’…. But yeah it’s there to have to know about it. GP21 I don’t think there’s much to lose with the test apart from people being anxious about their results. GP28 I really think it’s going to be better off offering it before pregnancy. Because once they are pregnant and they learn they have a double abnormal gene, then they are going to be really worried through the pregnancy, so I think it would be better before pregnancy. GP23 Is it going to cost them is a big issue, because you might think, great, we’ve got some news for you, you both are carriers for this, but now we would recommend IVF and pre-genetically testing that’s going to cost you a fortune. Is that a positive? Depends on the social circumstance of that family. MW04	**Emotional motivators** TDF: Emotion	For me, it’s that I feel I am making a difference. Mainly again because of this personal experience of this family who are going through this whole journey of a child with SMA [Spinal Muscular Atrophy]. GP24
		**Motivated by the value of RGCS for family planning** TDF: Beliefs about Consequences	It is like a huge area of potential benefit for couples long- term that should be accessible and easy to offer. GP27
		**Motivated by improving equity of access** TDF: Goals	It’s [MM] complementary to what we were doing anyway, so I just thought it was a great opportunity to increase access to our patients. GP05

**Table 3:** Detailed summary of Target behaviour 2: Identifying eligible patients.

Opportunities to identify eligible patients				
Barrier		Exemplar quotes	Enabler	Exemplar quotes
**i Environmental factors (part-time workload, patient cohorts and low consumer awareness/demand)** TDF: Environmental Context and Resources		I was only working a few days a week; therefore, my appointments were getting crowded-out by my regulars. So, the person who is going to the doctors to ask ‘hey, I was just wanting to know if there is anything I should be doing before I try to get pregnant?’ or the person presenting saying ‘I’ve got a positive pregnancy test’ couldn’t get into me very easily. GP14 My clientele seems to be the rusted on 85- to 92-year-olds - don’t have much pregnancy counselling there. GP21 There’s men as well doing it, but again women self-select women for women’s health, men self-select men for men’s health. So maybe 80/20, not that they don’t see pre-pregnant women and offer the testing, but they don’t see as many. GP13 I haven’t been able to offer that [RGCS] to the women because they don’t come in, it’s a very private…it’s women’s’ business in the refugee group. SHNurse20	**Intra-practice referral** TDF: Environmental Context and Resources ECR	I gave two of the other young female GPs the information. They basically said ‘Oh it looks great but we will send them to you’. So that was sort of meant to happen… then I suspect they forgot about it. GP14 Working part-time it is really hard, but because we have got nurse coverage over the five days, if there was a referral, I would have missed it and it would have gone to one of the other team members. SHNurse20
			**Clinics advertising or promoting which HCP to see for pregnancy planning** TDF: Environmental Context and Resources ECR	If I could put ‘If you’re planning on getting pregnant in the next 6 months consider talking to Dr…’ because I wasn’t seeing the volume of patients to pick them up by chance. GP14
			**Seeing patient pre-conception** TDF: Environmental Context and Resources ECR	I guess the ones you would like to see are the ones who are not actively planning a pregnancy, but they might at some stage, because by the time they are pregnant… it’s preferable to do it earlier. GP15
			**Rasing public awareness (i.e., campaigns)** TDF: Environmental Context and Resources ECR	You always have that conversation, but you often don’t have the conversation in the right timeframe. I’m often aware that patients are, between children if you like, but they don’t always disclose that they’re actively trying to have a baby. I guess perhaps it’s something about marketing or advertising in a wider sense outside of the practice setting so that people are aware of it. GP03
**ii HCP missing opportunities to offer RGCS** TDF: Memory Attention and Decision Making	I think I missed a couple where I took the Implanon out and didn’t think to ask ‘hey would you be interested in’ mainly because I was running two patients behind and the practice management might kill me. GP14 It’s pretty much just remembering to mention it amongst the myriad of other things that you have to deal with that the patient has actually come in for, so unfortunately sometimes it is a little bit of a second thought. GP11		**Making offering RGCS part of routine practice (antenatal and general visits)** TDF: Intentions	It’s pretty much routine in my brain that pre/early pregnancy consult I talk about it…It’s just on my brain stamp with the list of things I cover. GP28 It’s about raising it as a common question, it’s another tick box of saying ‘have you had your cervical screening done? Have you thought about having carrier screening?’ SHNurse20 I have caught quite a few discussing it with cervical screening because if you have got someone who is young, you can start to mention it if you know they have not had kids yet as well. So, there are other times not just around pregnancy where you can start discussing it as well. GP23 We have a lot of same sex couples here, so I do ask them if they know the donor because sometimes they do know the donor and it’s still a possibility for them to have the testing. GP10
			**Patient mentions they are planning a pregnancy** TDF: Memory Attention and Decision Making	Some people do come in talking about that they want to plan a pregnancy in the next 6 months and what should they do and that’s a lot easier to bring it up then and talk about it. GP30
			**Pre-planning which patients might be eligible** TDF: Behaviour Regulation	I do a bit of a handover on the families coming in for that clinic, and I’ll look at their history and their situation and say, ‘I think we should target these women - these are appropriate.’ MW04
			**Visual prompts to remind HCPs** TDF: Environmental Context and Resources	The website is quite easy to navigate and access, so I’ve found that I tend to have it in the background just as a reminder. GP11
			**Organising a return visit** TDF: Behaviour Regulation	Unfortunately, sometimes it is a little bit of a second thought. I have actually called patients back…and patients don’t seem to mind that. GP11
**iii HCPs being mindful of patients’ personal situation** TDF: Beliefs about Consequences	There are some that I just won’t mention it at all, I just think it’s too much for them [women seen in early pregnancy assessment clinics]’ GP22 Sometimes it’s a bit too much for them [migrant populations], and then they take away all the information and they don’t do anything about it, so rather than putting somebody unnecessarily through so much turmoil, we can pick and select our patient population. GP06 Less and less but sometimes we do see their religious beliefs do not allow them to pursue science in a way we would like them to…So if there is a clear understanding that it is going to be a no, then I would not offer it. CG16		**Building patient rapport** TDF: Skills	There are some where there is more than one consultation, I might mention it at the final consultation like ‘just to let you know this is something that is available should you be interested.’ GP22 A majority of the time I’ve met them on the wards, so I’m not an unfamiliar face…and I’ve already built some sort of rapport with them, and I can sit down with them and say, ‘look, if you are planning future pregnancies this is something that you might want to consider for yourself.’ MW04

**Table 4:** Detailed summary of Target behaviour 3: Offering RGCS to patients.

Providing pre- and post- test counselling			
Barrier	Exemplar quotes	Enabler	Exemplar quotes
**i Low consumer awareness or understanding of RGCS** TDF: Environmental Context and Resources	Most people have no idea what [RGCS] is when I bring it up. They always go, ‘isn’t that the Down syndrome testing or the harmony [NIPS]?’ GP07	**HCP building consumer awareness** TDF: Social Professional Role and Identity	Most patients don’t know anything or much about it, so it’s a good opportunity to talk about that it’s something available now if they are interested in doing that. GP29 People are very receptive to the message from us [GPs] and I think the same with this sort of genetic screening, I mean people know about it, but I think we are very trusted to encourage people to go through and get the testing done. GP01
		**Raising public awareness (campaigns, programmes like MM)** TDF: Environmental Context and Resources	It comes down to that poor time allocation we get given with our client face-to-face and therefore it needs to be raised at that social media level, whether it be radio, flyers, Facebook whatever format you can think of. SHNurse20 Raising the profile [through MM] has been helpful, I think it has made having the conversations easier. Among my colleagues as well as consumers and among midwifery staff. GP25
		**Word of mouth** TDF: Social Influences	It’s awareness and whether they have got friends that have done the same test before. I think a lot of people now rely of social media and their friends to tell them…or convince them rather than us health professionals. GP24
**ii HCP concern about pre- test counselling ability** TDF: Beliefs about Capabilities	Because you don’t know what sort of questions or queries are going to come up from the patient’s end…So when you might have two patients every couple of weeks who comes in for family planning it can be difficult to remember all the details. GP11	**Resources for HCPs (GC support and referral pathways to genetic services)** TDF: Environmental Context and Resources & Social Professional Role and Identity	I took notes while she [the GC] was talking. And I still refer to those little dot points that I made when I’m educating patients themselves. I obviously want to make sure that I’m giving them the right information and making it easily it’s easily digestible for them. GP27 Sometimes certain questions that they ask is beyond our knowledge skill, and then that’s when we have to call upon the genetic counsellor to come in and give their advice. GP06
		**Seek assistance from peers** TDF: Social Influences	If I know one of the doctors in the practice is seeing younger people and doing something I would probably ask them just because they are very accessible and easy. Or otherwise, a trusted senior colleague, one of the obstetricians of something like that. GP21
		**Confidence through practice** TDF: Skill	The more you do it, the easier it gets. GP11 I’ve been able to develop my own very simplistic way of getting the information across. MW08
**iii HCP concern about post-test counselling ability** TDF: Beliefs about Capabilities	I think the main thing would be concerns from the GPs about having to possibly counsel someone if they had a high-risk result. GP02	**Access to GC support** TDF: Environmental Context and Resources & Social Professional Role and Identity	As long as it’s made very clear that if someone does have a high-risk result, they are automatically referred to genetic counselling, I think that would take a lot of the GP concern away. GP02 Having good genetic counselling back-up, is the really important thing because as I said I can’t do that. Because it is actually really difficult work and really time-consuming work to do and it is very hard to fit it in when you are not an expert. GP15
**iv Time constraints in consults (antenatal and general visits, and for CALD patients)** TDF: Environmental Context and Resources	With the antenatal appointments that’s a massive appointment and it’s hard to get through all the stuff…There are a lot of different things that are available and it’s not much time to discuss what each of them provide and if they wanted to do those tests or not. GP29	**High consumer health literacy** TDF: Environmental Context and Resources	When I start screening I say, ‘do either of you work in health fields? or have you done grade 12 biology?’, and if someone pipes up and says ‘yes’, I’m like excellent, (laughs), because I know they’re going to get it straight away. GP13
	I would say time because you’ve got to do a mini genetics lesson in the middle of your consult…I can’t actually practice a good standard of medicine in six minutes, so, clearly time is a limiting factor in a lot of general practitioners’ lives. GP13	**Financial incentives or reimbursements** TDF: Environmental Context and Resources	if we could find any way to increase GP antenatal care [billing] that would be really good, because we do get it hard there. GP18
	I do know that for some families when they do go onto the website, they do find it difficult to navigate because of the language barrier, then I try to spend more time explaining the project, sometimes because of time limitations it is not possible. CG16	**Having RGCS offered pre-conception** TDF: Environmental Context and Resources	I find the first antenatal visit is a very long consult in itself, so having had the Mackenzie’s Mission or the carrier screening discussion done or declined, either way, it’s sort of that one less thing you have to do at the first antenatal visit. GP07
	There’s certainly things about the Mackenzie’s Mission website - I don’t know whether it’s available in other languages - and yeah so I think that would be a barrier. But I would still tell them to come and see me and sit down at the computer and translate it for them with the translator. MW08	**Simple and supported process for HCPs** TDF: Environmental Context and Resources	It’s time consuming to have the discussion, and the nice thing about Mackenzie’s Mission is I can talk about it as an option…but if they’re interested I can send them to your website and they can get a lot more information from that. GP15
			The other thing that I really like is the way it’s all done online, you don’t actually have to spend a lot of time talking about potential outcomes of carrier screening… And that will be really attractive to non-genetics professionals, there’s no way a GP can do carrier testing in 15 min GC09
			In terms of ease, it is just trying to fit it in with whoever comes in and sometimes when you are time pressured it can be difficult, but I found that not to be the challenge because I am there just to provide them that link and the information and ask them to go away and think about it. I give them a number [for a GC] if they want to ask any questions about it. GP19
		**Staging the offer process** TDF: Behaviour Regulation	I tell them about it, get them to go and have a look at the website, and then get them to book an appointment and we talk about it as a separate consult. I always wanted to make sure that their partner was on board as well before going ahead. GP01
		**Resources that help explain RGCS (website, diagrams, videos, auto fill request forms)** TDF: Environmental Context and Resources	I’ve got the 30 min time allocation with the client and I’m trying to fit in MM so I actually handed them my phone and showed them the little video, and they could consume that information while I would be setting up for an examination. I felt that was a better recruitment process than me trying to repeat myself with every potential client. SHNurse20 I find the time is one big factor and I have developed some diagrams which I keep with me. I find diagrammatic representation much easier than talking through these things. Especially for the population who are coming from an English-speaking diverse background. CG16 It’s always nice to have a form that works with whatever software the GP’s using, [the form] is auto-filled and then that record is still in that patient’s notes when we’ve sent it. I think if this was going to roll out, that needs to happen. GP05
		**Well-informed about RGCS** TDF: Knowledge	You want to be fully informed on the concept in the first place, so that it makes the consult effective, quick, the information is there, questions can be answered easily without trying to look things up. GP07
**Concerns around regional accessibility** TDF: Environmental Context and Resources	My big thing being rural is, I grew-up in Melbourne where you have great access to services but coming to [a regional town] I realised there is a lack of access to opportunities. SHNurse20	**Self-collect samples and telehealth** TDF: Environmental Context and Resources	I do telehealth sometimes with countrywomen…so I’m going to try and incorporate more into the country women there, and it’s something that I would have to post out to them. I don’t know whether that would have any issues, it shouldn’t do because the packs are sent out and it’s self-doing. In theory it shouldn’t, I just have to post the paperwork out to them. MW04
		**Dedicated outreach services** TDF: Environmental Context and Resources	I think we are going to have to develop strong community programmes, whether that is at school…or we have breast screen vans rolling into community and whether we do this with genetic carrier screening to create awareness. CG17

Perceptions of patient receptivity towards RGCS			
**i Financial barriers** TDF: Environmental Context and Resources	The barrier in the past has always just been cost because it was so expensive. GP01 Cost is probably a big thing. I have been surprised by peoples probably lack of interest in it, and then if they have to pay $600 on top of that, I think it would be quite limited. GP30	**Funding for RGCS** TDF: Environmental Context and Resources	It’s probably unreasonable for it to be this great, like free and testing so many things but I think there would be really wide uptake if it was. I think people have to pay for it even a little bit it won’t be as popular. GP28 …part of the cost should include the genetic counselling, I don’t think it should be an extra. So, everyone pays X amount for it but that includes the genetic counselling for those who need it. GP15
**ii Patient beliefs** TDF: Environmental Context and Resources	Sometimes religious beliefs come into play. I see populations and certain ethnicities who would not want to go ahead with these sorts of things. CG16 It’s [culture] so ingrained, especially the first-generation migrants, sometimes that trumps medicine, so we can go blue in our face explaining over and over again … ultimately, it’s just respecting their beliefs and trying to make the best out of it and ensuring a positive outcome. GP06	**Framing conversations around the benefits of knowing carrier status** TDF: Skill	Sometimes there are people like them who are worried because in Islam we don’t do a termination very easily…but I said ‘look even if you don’t go for a termination, it sets you up so that you are aware of what happens afterwards’. GP18 I might mention that so it’s not necessarily that you would terminate the pregnancy but you might choose to learn a bit about the condition, to talk to specialists, to join a support group, peer information and that sort of thing. GP25
		**Patients taking up the offer** TDF: Reinforcement	All but two people I’ve discussed it with have ended up taking up the opportunity, which is really good. GP26
	The reason people chose not to was they either came from a faith tradition where that was not something they wish to enquire about or even if they didn’t have a faith background tradition, it was just they didn’t know what they would do with that information and they were aware it was going to create angst and issues for them. GP25	**Sense of reward for offering RGCS** TDF: Reinforcement	It’s fantastic, everyone’s been excited about do it, in fact one of my couple’s is already pregnant, have just come in today because they’re pregnant, so that’s very exciting. GP01
**iii Not knowing when patient’s take-up testing** TDF: Reinforcement	I don’t know who actually decides to do it until I get their result, however long later. GP02	**Altered when patient supplies a sample** TDF: Reinforcement	We know how many forms we’ve given out and slowly the results trickle in but getting an idea of how many of your patients have taken up the screening is nice to know. GP07

### Target behaviour 1: Engaging with RGCS

Two overarching barriers were identified: *awareness, understanding and education about RGCS* and *HCPs’ attitudes towards and beliefs about RGCS*. Figure 2 and Table 2.

*Awareness and understanding of RGCS:* Three barriers were associated with a lack of, or low knowledge of, RGCS within primary health care.

i. **Low awareness of RGCS (TDF: Knowledge)** especially compared with other antenatal tests (e.g., [NIPS]). Enablers included promotion by their peers, professional bodies, and their patients raising RGCS (TDF: Social Influence).
ii. **Low understanding (TDF: Knowledge)** of RGCS was reported by many HCPs. Education sessions that allowed HCPs to ask questions were cited as a helpful way to improve understanding. (TDF: Knowledge and Social Influences).
iii. **Time/logistics of participating in education (TDF: Environmental Context and Resources)**, was found to be time consuming, taking away from direct patient contact, though enabled by receiving professional development points (TDF: Reinforcement) or making education available through different modes (TDF: Environmental Context & Resources).

*HCP attitudes towards and beliefs of RGCS* (TDF: Social and Professional Role and Identity): Two barriers were identified in this overarching barrier.

i. **Lack of HCP interest (TDF: Social and Professional Identity)**, for some HCPs this is attributed to not seeing the relevant population of patients though others did not feel offering screening was part of their role. Enablers include inter-practice referrals (TDF: Environmental Context and Resources).
ii. **Concerns about negative effects (TDF: Belief about Consequences)** e.g., patient anxiety about screening or financial implications for families who receive an increased chance result. However, emotional connection acted as an enabler (TDF: Emotion).

### Target behaviour 2: Identifying eligible patients

One overarching barrier was identified in this target behaviour, *opportunities to identify eligible patients*. Figure 2 and Table 3.

#### Opportunities to identify eligible patients

Three barriers were apparent within this overarching barrier: environmental factors (TDF: Environmental Context and Resources); forgetting to offer (TDF: Memory Attention and Decision Making); and patients’ personal situations (TDF: Belief about Consequences).

i. **Environmental factors (TDF: Environmental Context and Resources)**. One HCP reported they would be unlikely to see a patient (especially a female patient) in a reproductive healthcare context, a sentiment shared by some of the female GPs when speaking about their male colleagues. Others were concerned about when to time making the offer and questioned whether early pregnancy was an appropriate time. Several participants reported initiatives to raise awareness of RGCS could function as an enabler (TDF: Memory Attention and Decision Making) to prompt a change in patients initiating the conversation with their HCP.
ii. **Forgetting to offer (TDF: Memory Attention and Decision Making)**. Missed opportunities to identify patients were commonly cited, especially HCPs forgetting RGCS due to competing priorities. Most participants spoke about making RGCS a priority in their practice, so that offering RGCS becomes part of routine practice, including for same sex couples (TDF: Intentions). Some incorporated offering into other screening visits (TDF: Behavioural Regulation) such as cervical screening.
iii. **Perceptions of patients’ personal situations (TDF: Belief about Consequences)**. Some HCPs were mindful about raising anxiety for women they see in early pregnancy assessment clinics or ensuring they take patient’s cultural beliefs into consideration. Some reflected on their messaging to be clear that preparing if a child may require early intervention or have special needs can be helpful. Being able to build rapport with patients through continuity of care allowed HCPs (especially midwives) to judge each situation on a case-by-case basis (TDF: Skills) and ensure RGCS was offered, or even just mentioned as an option to patients regardless of their situation.

### Target behaviour 3: Offering RGCS to patients

Two overarching barriers were identified in this target behaviour, *providing pre- and post- test counselling, and patient receptivity*. Figure 2 and Table 4.

#### Providing pre- and post- test counselling

HCPs reported four interrelated barriers to counselling during these phases.

i. **Low consumer awareness (TDF: Environmental Context and Resources)**. HCPs reported low consumer awareness of RGCS and patient confusion with other prenatal tests (i.e., NIPS). Consequentially, HCPs described a sense of responsibility to take the time to increase awareness and explain in an easily digestible way what RGCS involves and considered themselves well placed to at least raise the topic (TDF: Social and Professional Identity).
ii. **Concern about ability to counsel (TDF: Belief about Capabilities)** were reported by some HCPs especially when not regularly providing pre- and early pregnancy care including, mixing up RGCS and NIPS or raising potentially worrying or distressing information. Genetic counsellors were favoured as a resource for HCPs to seek guidance, others sought peer advice (TDF: Social Influences), and some felt practice was key (TDF: Skills). Particularly for post-test counselling, HCPs reported they valued having access to genetic counselling support as needed for both themselves and for couples (TDF: Environmental Context and Resources and Professional Role and Identify). HCPs who had patients take up testing felt more confident in their abilities and in some cases felt rewarded, experiencing patient gratitude to be offered RGCS.
iii. **Time constraints (TDF: Environmental Context and Resources)** were a dominant challenge. Coupled with the previous barriers HCPs were concerned about the time to inform patients about RGCS. HCPs found the Mackenzie’s Mission website helpful in this situation, when there were time constraints they would provide the website and encourage patients to review the content in their own time (TDF: Environmental Context and Resources).
iv. **Regional barriers (TDF: Environmental context and resources)**. HCPs consider the Mackenzie’s Mission model facilitated couples living in regional and remote areas acceptable access and was successfully offered via telehealth. Outreach services providing education and offering RGCS was cited as a model that could be used in addition, to reach communities with limited access to healthcare (TDF: Environmental context and resources).

#### Perceptions of patient receptivity

HCPs perceived three barriers in relation to patients’ receptivity to RGCS.

i. **Financial barriers (TDF: Environmental Context and Resources)**. Cost was identified as a previous barrier to offering testing, and looking to the future, HCPs felt funding should cover GCs counselling for increased chance couples (TDF: Environmental Context and Resources), whereas the additional services included in Mackenzie’s Mission (e.g., one round of IVF) whilst generous were considered unlikely to be sustainable.
ii. **Patients’ religious and ethical beliefs (TDF: Intentions)**. Patients’ religious beliefs, or patients with ethical concerns around termination of pregnancy were discussed by some HCPs. In most circumstances, HCPs felt they could still provide patients with the information that allowed them to make an informed choice in line with their values, framing the conversation around the benefits of knowing your carrier status (awareness and preparedness, becoming knowledgeable about the condition, talking to specialists, joining support groups etc.) (TDF: Skills).
iii. **Not knowing whether the sample has been supplied (TDF: Environmental Context and Resources)**. Some HCPs ant to be made aware when a couple provided a sample to the laboratory for screening to accommodate follow-up appointments. Here, HCPs could follow-up with the patient if required (TDF: Reinforcement).

## Discussion

To support any change in a behaviour or activity, such as offering RGCS, it is essential to clearly specify the target behaviour(s) required and the associated barriers and enablers at each stage. Without this understanding there is a risk of investing resources to design solutions for potentially non-existent problems, wasting time and effort [26]. This study investigated the experiences of HCPs, with particular focus on non-genetic professions, offering population RGCS and categorised findings into three sequential target behaviours: 1. Engaging with RGCS, 2. Identifying eligible patients to offer RGCS to, and 3. Offering RGCS to patients. We identified 15 associated barriers and significantly more enablers (*n* = 44) which could reflect the nature of the participants, who as early adopters are often positive about the change and are understood to influence the behaviour of those around them by making the behaviour change more observable [27]. Indeed, **social influences** were shown in this study to be a principal factor in HCPs’ initial engagement with RGCS and increasing awareness amongst their peers and will play a key role in the success in the roll out of future population RGCS programmes. Acknowledging previous research undertaken in this area, here we discuss HCPs’ preferred option for offering RGCS through a comprehensive model of care, like the approach taken in Mackenzie’s Mission, the associated challenges and identify implications for future service delivery. TDF domains are indicated with **bold text**.

The Mackenzie’s Mission model of care required HCPs to provide the offer of testing and direct the couple to a study participant portal. There the couple were provided with education and a decision aid to make informed decision about RGCS. Although this approach was designed to minimise the time taken for HCPs to offer RGCS, it meant HCPs lacked **knowledge of the patients’** journey and did not know whether the patient had in fact accepted the offer of screening. Understanding their role in the process appeared key to HCPs improving **belief about their capabilities** in incorporating this more ‘hands off’ way to offering RGCS into their practice. Some HCPs hoped the model, especially the online education and consent which is favoured for overcoming the complexities of consent in genomic medicine [28], would continue when RGCS becomes more widely accessible.

Equity was raised by many participants. We did not capture data on HCP ethnicity which may have provided a useful lens with which to analyse some HCP comments and assumptions on when to offer RGCS or not. Some HCPs expressed concern about when it would be appropriate to offer screening for some communities e.g. migrant populations. There is the risk of unconscious bias acting as a barrier to equity of access through the offer, or lack of to RGCS. Whilst no access to technology barriers were reported, several HCPs raised concerns about **potential consequences due to** language barriers for couples from culturally and linguistically diverse backgrounds being able to access online material without HCP assistance [29]. The availability of online translation services may help overcome these concerns [30]. Overall, the ease of access was found complimentary to telehealth and an acceptable way to offer RGCS for regional/remote areas. The model also worked for donor couples, where the donor could provide a sample, allowing HCPs to be able to offer equitable care.

Accessing RGCS through Mackenzie’s Mission still requires HCPs to have the **knowledge** and **skill** to identify patients for whom RGCS is appropriate. Within the Mackenzie’ Mission programme an extensive amount of work has been conducted and reported on the role (and design) of education in the implementation of genomics and large-scale carrier screening programmes. Despite widespread consensus that RGCS is best situated in primary care, aligning with HCPs’ **professional identity**, and offered pre-conception [2, 7, 31], research shows higher uptake among pregnant women [32]. Not only does offering pre-conception allow couples access to greater reproductive options, but our study also indicates offering in pre-conception lessens other HCPs barriers (e.g., **environmental** time constraints in antenatal appointments, and HCPs concern about the **potential consequences** for patient anxiety). However incorporating the offer of RGCS into pre-conception care (public funding capped at 40 min*)* appeared to be more challenging, due to **a lack of resources** and **forgetting to offer** due to competing priorities in short appointment sessions with HCPs finding it easier to discuss RGCS when the conversation is initiated by the patient [9]. This lack of time will remain a key challenge and identifying mechanisms to support HCPs will be essential to drive successful take up and implementation of future RGCS programmes. Undoubtedly, raising community awareness of RGCS as a part of pre-conception care is needed to facilitate greater patient receptivity and ability to make informed decisions about screening. As patient health literacy was considered an enabler for HCPs to offering RGCS, increasing consumer awareness may also improve equity of access. HCP **skill** and **intentions** to incorporate RGCS into general practice appointments was an attributable factor to opportunistically identifying pre-conception patients. Although HCPs found raising RGCS in early pregnancy easier, some HCPs expressed **concern about the consequences** with the potential of upsetting the couple during this period. Fears of medicalising pregnancy have previously been identified [13, 33] and additional tools (e.g., decision aids [34]) are required to ensure couples can make decisions that align with their values [32].

Unlike the other target behaviours, HCPs did not recognise any Mackenzie’s Mission specific supports provided for the second target behaviour, identifying eligible patients. Where enablers are lacking, often in more complex areas (e.g., forgetting to offer RGCS pre-conception—TDF **Memory Attention and Decision Making**) the application of the behaviour change theory, through coding with the TDF [35], can offer additional theory informed behaviour change techniques using the Theory and Techniques Tool [https://theoryandtechniquetool.humanbehaviourchange.org/] [36] to support HCPs. For example, theory informed behaviour change techniques aligned with Memory, Attention and Decision Making include ‘*prompts and cues*’, e.g.,, setting a reminder on the GP information system to prompt the ‘One Key Question®’ [37] discussion with all patients of reproductive age to ask if they are planning a pregnancy in the near future. If yes, the HCP can share a range of health considerations including the option of RGCS.

One reported area of concern was the need for counselling couples who receive a 1 in 4 chance of affected children with **HCPs’ belief about their capabilities**. The Mackenzie’s Mission model ensured a study genetic counsellor was available and they played a critical role in supporting HCPs with expert knowledge and skills, and also couples, as they made reproductive decisions to align with their values. Further provision of RGCS through primary care will require careful consideration of how genetic counselling services could be provided [34, 38, 39]. Given HCPs perception of their own ability of offer RGCS appears to be contingent on availability of expert genetic counselling and clinical support, it is essential that population-based programmes provide this support.

**Fig. 3:**
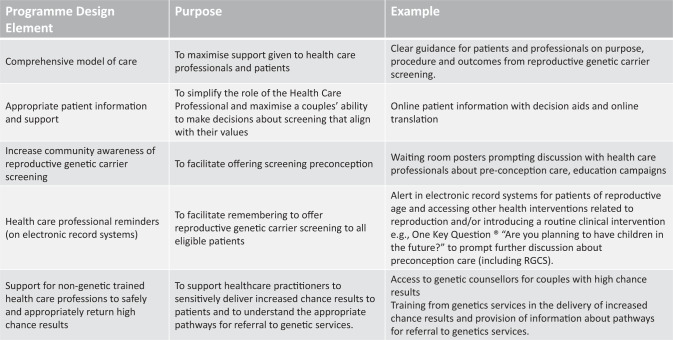
Examples of aspects of programme design that can contribute to reproductive genetic carrier screening being more accessible.

In addition to HCP individual level experiences noted here, future implementation of national RGCS programmes while dependent on local context [40] may benefit from aspects of programme design. Figure 3 reports several features identified from this study that can contribute to making reproductive genetic carrier screening more accessible.

### Limitations

Drawing on the experience of early adopters is likely to have introduced positivity bias, with the possibility that HCPs who were likely to respond to interview invitations were those most likely highly engaged with Mackenzie’s Mission. Mitigating that, a third of participants had no previous experience with RGCS. Of the 168 potential participants we only interviewed 31 either due to lack of response or the HCP was unable to participate. We were also unable to capture the views of those who declined to participate in the Mackenzie’s Mission study. The SARS-CoV-2 pandemic led to data collection taking over a year, which was longer than planned, meaning some external contextual factors may have changed. This study was undertaken in the context of an Australian Government funded research project where generalisation may be limited to Australian health system and a well-resourced research project (under Mackenzie’s MIssion, RGCS is offered free of charge to the couples, genetic counselling is offered to couples receiving a high-chance result and to support HCPs so may not reflect other health systems or state-wide or national RGCS programmes.

At present, RGCS is offered in an ad hoc manner and access is variable. Whilst population-based approaches will make access more equitable, targeted support for HCPs to offer patients the option of RGCS is required. Behaviour change theory provides a structured approach to learning from the experience of early adopters and an opportunity to identify the determinants influencing implementation. The key steps of collection and coding of barriers and enablers by each target behaviour identified outlined here can now be used to select and test theory informed implementation strategies. Although this study shows HCPs’ strong preference for offering RGCS through a comprehensive model of care, and various other programme design elements that reduce barriers, it is essential that future research continues to leverage behaviour change theory to develop and test programme design elements that contribute to RGCS being provided in an equitable and accessible way.

## Supplementary information

File 1 and 2

## Data Availability

The datasets generated during and/or analysed during the current study are available from the corresponding author on reasonable request.
