# Supplementary material for: The more you do it, the easier it gets: using behaviour change theory to support health care professionals offering reproductive genetic carrier screening
Source: Eur J Hum Genet. 2022 Nov 24;31(4):430–44. doi: 10.1038/s41431-022-01224-5 (PMC9686264; doi:10.1038/s41431-022-01224-5)
Supplement: Supplementary file 1 — File 1 and 2 [file 41431_2022_1224_MOESM1_ESM.pdf]

Supplementary File 1: Semi-structured interview schedule guided by the COM-B Framework

Interview Structure

Firstly, I would like to find out a little more about your area and experience of genetic carrier screening

Interview Schedule

(C – capability, O – opportunity, M – motivation)

**Area of interest**	**Sample questions**	**Sample prompt**
**Context**	What clinical area to do you work in?	• GP, midwife etc
**Starting off/ maintaining involvement with Mackenzie's Misson**	Before Mackenzie's Mission, did you have any previous experience in offering genetic carrier screening? (C)<br><br>Why did you choose to participate/not participate in Mackenzie's Mission? (M)<br><br>Are any of your colleagues participating in Mackenzie's Mission? (M)<br><br>Starting off was there anything that would have made participating in Mackenzie's Mission easier? (O)<br><br>*(for participating HCPs)* Now Mackenzie's Mission is underway, is there anything you find challenging about offering genetic carrier screening (C, O, M)<br><br>Have any of your patients decided not to participate? What are the reasons they give?<br><br>Or is there anything you find helpful when thinking about offering genetic carrier screeninh? (C, O, M)	• Maybe directing couples to funded opportunities, previous training, interest<br><br>• Either collocated or working in other clinical areas<br><br>• Is there anything that prompted you (either way)?<br><br>• GC support, more time, understanding results, knowing where to send high risk couples for advice<br><br>• Reasons: practical, don't want to be in research, don't want genetic screening, too many other things going on.<br><br>• Skills, resources, observing others
**Looking to the future**	How do you feel genetic carrier screening should be provided in the future? (C, O, M)<br><br>Do you see it as your profession's role? (M)<br><br>Who should fund genetic carrier screening? (O)	

Close: Are there any other areas that we haven't discussed, and you would like to share?

Thank you for participating. If any other areas occur to you later please feel free to get in touch with me.

Supplementary File 2: COM-B Framework and Theoretical Domains Framework definitions and definitions in context coding guide

COM-B codes	TDF domain	TDF domain definition (Cane et al. 2012)	Definition in context
Capability (Psych & Physical)	Knowledge	An awareness of the existence of something	HCPs’ and peers’ actual awareness and understanding (through education/training) of the existence, guidelines, principles and process of offering RGCS
	Skills	An ability or proficiency acquired though practice	HCPs’ actual physical and psychological ability or proficiency acquired through actual practice (as opposed to education/training – cannot get skills through education) to make decisions whether or not to offer patients RGCS or ability to offer RGCS.
	Memory, Attention and Decision Processes	The ability to retain information focus selectively on aspects of the environment and choose between two or more alternatives	HCPs’ ability to remember to consider RGCS alongside other pre-conception or early pregnancy routine care, or other relevant procedures (contraception discussions) or their decision-making process around discussing RGCS with a patient
	Behavioural Regulation	Anything aimed at managing or changing objectively observed or measured actions	HCPS’ self-created or self-imposed, or organisations regulation to help make decisions about offering RGCS.
Opportunity (Social & Physical)	Social Influences	Those interpersonal processes that can cause individuals to change their thoughts, feelings, or behaviours	Interpersonal interactions between professionals or consumers that can influence HCPS’ thoughts, feelings or behaviours (ie anything in Motivation) regarding the use of RGCS
	Environmental Context and Resources	Any circumstance of a person’s situation or environment that discourages or encourages the development of skills and abilities independence, social competence and adaptive behaviour	Any external circumstance of a HCPS’ situation or environment that HCPs consider discourages or encourages them to offer RGCS in practice, including impacting the development of capability, motivation or social opportunity to offer RGCS
Motivation (Automatic & reflective)	Social/Professional Role and Identity	A coherent set of behaviours and displayed personal qualities of an individual in a social or work setting	HCPs’ perceived professional role and identity in relation to offering and informing patients about RGCS
	Beliefs about Capabilities	Acceptance of the truth, reality or validity about at ability, talent, or facility that a person can put to constructive use	HCPs’ perception about their own capability to offer RGCS (terms used in literature: confidence, comfort, control)
	Optimism	The confidence that things will happen for the best or that desired goals will be attained	HCPs’ optimism or pessimism around the use of offering RGCS and that RGCS will have a positive impact on couples access, reproductive options knowledge and family planning
	Beliefs about Consequences	Acceptance of the truth, reality, or validity about outcomes of a behaviour in a given situation	HCPs’ perceptions about the value of offering RGCS– whether it is worthwhile in that it will improve patient outcomes (term used in literature: attitude)
	Intentions	A conscious decision to perform a behaviour or a resolve to act in a certain way	HCPs’ intentions to offer RGCS
	Goals	Mental representations of outcomes or end states that an individual wants to achieve	Whether offering RGCS is a priority within their clinical practice, something that they want to offer their patients

	Reinforcement	Increasing the probability of a response by arranging a dependent relationship, or contingency, between the response and a given stimulus	Incentives, rewards, sanctions, reinforcement at any level (e.g., patient satisfaction; better client health; economic incentives) that encourage or increase HCPS' decisions to offer RGCS
	Emotion	A complex reaction pattern, involving experiential, behavioural, and physiological elements, by which the individual attempts to deal with a personally significant matter	HCPs' feelings towards offering RGCS
